# Two out of one: revising the diversity of the epiphytic fern genus *Scleroglossum* (Polypodiaceae, Grammitidoideae) in southern China

**DOI:** 10.3897/phytokeys.130.33979

**Published:** 2019-08-29

**Authors:** Hong-Mei Liu, Jian-Yong Shen, Zhen-Long Liang, Feng Peng, Wei-Zhen Wang, Zu-Wei Yang, Shuang Wang, Barbara Parris, Harald Schneider

**Affiliations:** 1 CAS Key Laboratory of Tropical Plant Resources and Sustainable Use, Xishuangbanna Tropical Botanical Garden, Chinese Academy of Sciences, Menglun, Yunnan 666303, China; 2 Xishuangbanna Tropical Botanical Garden, Chinese Academy of Sciences, Menglun, Yunnan 666303, China; 3 Centre for Integrative Conservation, Xishuangbanna Tropical Botanical Garden, Chinese Academy of Sciences, Menglun, Yunnan 666303, China; 4 Forestry College of Guangxi University, Nanning, Guangxi 530005, China; 5 Tongbiguan Provincial Nature Reserve, Dehong, Yunnan 678400, China; 6 Fern Research Foundation, 21 James Kemp Place, Kerikeri Bay of Islands 0230, New Zealand

**Keywords:** Cryptic speciation, DNA barcoding, grammitid ferns, Yunnan

## Abstract

Our understanding of the flora of China has greatly improved during the last 100 years but effective management of the rich biodiversity and unique natural resources requires resolving the taxonomic limitations of existing treatments. Here, we focus on the epiphytic genus *Scleroglossum* with special emphasis on the occurrences in Hainan and Yunnan of mainland China. By combining fieldwork, herbarium studies, and DNA barcoding we test the hypothesis that this genus is represented by more than one species in China. Our integrative results show the Yunnan accessions are distinct from those in Hainan in both phenotypic and genotypic variation. The Yunnan accessions belong to *S. pusillum*, whereas the Hainan accessions represent a distinct species displaying the morphological characteristics of *S. sulcatum*. Genotypic evidence suggests the occurrence of cryptic diversity among accessions with the morphology of *S. sulcatum*. In summary, the study contributes to the crucial assessment of the plant diversity in Yunnan and illustrates the importance of integrating collection efforts and DNA barcoding approaches to enable effective assessment of the epiphytic diversity of Yunnan.

## Introduction

Grammitid ferns (Grammitidoideae, Polypodiaceae) are with ca. 911 species one of the most species rich lineages of ferns (PPG1 2016). These ferns are distributed throughout wet tropical habitats but their ranges expand into wet subtropical and temperate climate zones (e.g. Parris 2003, Ranker et al. 2004, Sundue et al. 2014, Bauret et al. 2017). Most grammitid ferns grow as epiphytes but some species prefer saxicolous or rheophytic habitats. The classification and taxonomy of these ferns have been challenging with the consequence that recent research integrating phylogenetic approaches resulted in a remarkable transformation of the generic classification of these ferns (e.g. Ranker et al. 2004, Sundue et al. 2014, Bauret et al. 2017, Parris 2018). A major challenge is the assessment of local species diversity as a consequence of a combination of factors including the frequent occurrence in remote areas, often restricted and disjunct distribution ranges, and homoplasy in many key-characteristics used to identify these plants (e.g. Parris 2003, 2018, Ranker et al. 2004). The mostly elusive morphological differentiation between closely related grammitid ferns requires careful investigation of the often few available specimens by researchers with special taxonomic expertise. Thus, assessing the threats to the grammitid fern diversity is a challenging but crucial task to enable the conservation of these unique ferns despite the rapid environmental changes of their habitats as a consequence of deforestation and global climate change. The application of DNA-barcoding approaches holds the promise to resolve the species identification issues but very little attention has yet been given to the application of these methods to assess and monitor the occurrences of these ferns. Several studies demonstrated successful application of DNA barcoding to clarify the distribution/ ecology of ferns (e.g. de Groot et al. 2011, Nitta et al. 2017) and species taxonomy (Shu et al. 2017) as long as reticulate evolution is taken into account (e.g. Wang et al. 2016, Liu et al. 2018). As a crucial step towards the application of DNA barcoding to monitor the grammitid diversity throughout China, we are required to re-assess the known occurrences and species diversity of these ferns in China.

Similar to other plant groups, our understanding of grammitid ferns diversity of China has been steadily improved. The treatment of these ferns in the Flora Reipublicae Popularis Sinicae (FRPS, Zhang 2000) recognized 22 species, whereas the Flora of China (FOC, Zhang et al. 2013) accepted 31 species. A recent study with special emphasis on Taiwan (Knapp and Hsu 2017) increased the number of species occurring in China to 35 species (see Table 1). Differences between the treatment provided in FRPS and FOC are not only restricted to the number of species recognized (9 species; 29%) and their generic classification but also to the improved understanding of the species identity with several species previously recorded using either synonyms (4 species; 13%) or wrongly applied names (5 species; 16%). However, some conflicts may require further attention. For example, treatments in both FRPS and FOC, agreed on a single species of the mainly tropical Asian genus *Scleroglossum* Alderw. occurring in China but differ about the accepted name. *Scleroglossum pusillum* (Blume) Alderw. was the name recorded for the Chinese occurrences (e.g. Zhang 2000) until the FOC treatment recognized instead *Scleroglossum sulcatum* (Kuhn) Alderw. and the authors argued the misidentification in FRPS (Moore and Parris 2013). These two species are morphologically distinct but frequently confused in older treatments. Both species show a wide distribution range in tropical SE Asia. The most recent treatment of this genus for Vietnam included the two above mentioned species as well as *S. pyxidatum* Alderw. (Parris et al. 2015). Besides, the two treatments (FRPS and FOC) differ in the reported distribution of the genus in China. The FOC treatment reported only Taiwan and Hainan as occurrences, whereas the FRPS treatment accepted a wider range of this species in southern China. Besides Hainan (Mt. Wuzhishan, Mt. Yinggeling) and Taiwan (North), the genus *Scleroglossum* was previously recorded to occur in the Nanling Mountains of Guangdong (Yan et al. 2007, Zhang and Li 2011), Daming Mountain and Shangsi County in Guangxi (Zhou and Li 1993, Qin and Liu 2010, Jiang and Liu 2014), and Mt. Laojunshan in Yunnan (Lu and Zhang 1994). Consequently, the Species Catalogue of China Vol. 1 (Yan et al. 2016) recorded one species of *Scleroglossum*, namely *S. sulcatum*, to occur in Guangdong, Guangxi, Hainan, Taiwan, and Yunnan.

**Table 1.** Summary of the grammitid ferns (Grammitidoideae, Polypodiaceae) diversity of China (Chinese species printed in bold). Columns report: "Genus" recorded genera according to PPG 1 (2016); "Species" recorded species occurring in China according to the most recent publications; "2000" species reported in Zhang (2000); "2013" species recorded in Zhang et al. (2013); "2016" species recorded in Yan et al. (2016); "2017" species recorded in Yan et al. (2016) plus Knapp and Hsu (2017) ; "TaxCon" consistency of species names used in the different taxonomic records (CO = same name used throughout, MI = misapplied species names in older treatments, NA = not applicable; SY = synonyms used in older publications); "PhyRes" species represented in the *rbcL* dataset (0 = absent, 1 = present)). The following columns report present (1) or absence (0) in Chinese provinces with grammitid records. ChPrSp reports the number of provinces in which each grammitid species has been recorded. The final column "Vietnam" reports occurrences in Vietnam.

Genus	Species	2000	2013	2016	2017	TaxCon	PhyRes	Anhui	Fujian	Jiangxi	Zhejiang	Guangdong	Guangxi	Guizhou	Hunan	Sichuan	Xizang	Yunnan	Hainan	Taiwan	ChPrSp	Vietnam
***Acrosrous* Copel.**	*A. friderici-et-pauli* (Christ) Copel.	0	0	0	0	NA	NA	0	0	0	0	0	0	0	0	0	0	0	0	0	0	1
***Calymmodon* C.Presl**	***C. asiaticus* Copel.**	1	1	1	1	CO	1	0	0	0	0	0	1	0	0	0	0	0	1	0	2	1
	*C. concinuus* Parris	0	0	0	0	NA	NA	0	0	0	0	0	0	0	0	0	0	0	0	0	0	1
	***C. gracilis* (Fee) Copel.**	1	1	1	1	CO	1	0	0	0	0	0	0	0	0	0	0	0	0	1	1	1
	***C. oligotrichus* T.C.Hsu**	0	0	0	1	NA	0	0	0	0	0	0	0	0	0	0	0	0	0	1	1	0
	***C. ordinatus* Copel.**	0	1	1	1	CO	0	0	0	0	0	0	0	0	0	0	0	0	0	1	1	0
***Chrysogrammitis* Parris**	***C. glandulosa* (J.Sm.) Parris**	1	1	1	1	SY	1	0	0	0	0	0	0	0	0	0	0	0	0	1	1	
***Ctenopterella* Parris**	***C. blechnoides* (Grev.) Parris**	1	1	0	1	SY	1	0	0	0	0	0	0	0	0	0	0	0	1	0	1	1
	*C. nhatrangensis* (Baker) Parris	0	0	1	0	NA	NA	0	0	0	0	0	0	0	0	0	0	0	0	0	0	1
***Dasygrammitis* Parris**	*D. brevivenosa* (Alderw.) Parris	0	0	0	0	NA	NA	0	0	0	0	0	0	0	0	0	0	0	0	0	0	1
	***D. mollicoma* (Nees & Blume) Parris**	1	1	1	1	CO	1	0	0	0	0	0	0	0	0	0	0	0	0	1	1	1
***Micropolypodium* Hayata**	***M. okuboi* (Yatabe) Hayata**	1	1	1	1	CO	1	0	1	0	1	1	1	1	1	0	0	0	1	1	8	1
	***M. sikkimense* (Hieron.) X.C.Zhang**	1	1	1	1	CO	1	0	0	0	0	0	1	1	1	1	1	1	0	0	6	0
***Oreogrammitis* Copel.**	***O. adspersa* (Blume) Parris**	1	1	1	1	CO	0	0	0	0	0	0	0	0	0	0	0	0	1	1	2	0
	***O. congener* (Blume) Parris**	1	1	1	1	CO	0	0	0	0	0	0	0	0	0	0	0	0	0	1	1	1
	***O. dorsipila* (Christ) Parris**	1	1	1	1	CO	1	0	1	1	1	1	1	0	0	0	0	0	0	0	5	1
	***O. hainanensis* Parris**	0	1	1	1	NA	0	0	0	0	0	0	0	0	0	0	0	0	1	0	1	0
***Oreogrammitis* Copel.**	***O. nuda* (Tagawa) Parris**	1	1	1	1	CO	0	0	0	0	0	0	0	0	0	0	0	0	0	1	1	0
	***O. orientalis* T.C.Hsu**	0	0	0	1	NA	0	0	0	0	0	0	1	0	0	0	0	0	0	1	2	0
	*O. parvula* Parris	0	0	0	0	NA	NA	0	0	0	0	0	0	0	0	0	0	0	0	0	0	1
	***O. reinwardtii* (Blume) Parris**	1	1	1	1	CO	1	0	0	0	0	0	0	0	0	0	0	0	0	1	1	1
	***O. sinohirtella* Parris**	0	1	1	1	NA	0	0	1	1	1	1	1	1	1	0	0	0	0	0	7	1
	*O. subevenosa* (Baker) Parris	0	0	0	0	NA	NA	0	0	0	0	0	0	0	0	0	0	0	0	0	0	1
***Prosaptia* C.Presl**	*P. alata* (Blume) Christ	0	0	0	0	NA	NA	0	0	0	0	0	0	0	0	0	0	0	0	0	0	1
	***P. barathrophylla* (Baker) M.G.Price**	1	1	1	1	MI	0	0	0	0	0	0	0	0	0	0	0	0	1	0	1	1
	***P. celebica* (Blume) Tagawa & K.Iwatsuki**	0	1	1	1	NA	0	0	0	0	0	0	0	0	0	0	0	0	0	1	1	1
	***P. contigua* (G.Forster) C.Presl**	1	1	1	1	CO	1	0	0	0	0	1	0	0	0	0	0	1	1	1	4	0
	***P. formosana* (Hayata) T.C.Hsu**	0	0	0	1	NA	0	0	0	0	0	1	0	0	0	0	0	0	0	1	2	0
	***P. intermedia* (Ching) Tagawa**	1	1	1	1	MI	0	0	0	0	0	1	1	0	0	0	0	0	1	1	4	1
	***P. nutans* (Blume) Mett.**	0	1	1	1	NA	1	0	0	0	0	0	0	0	0	0	0	0	0	1	1	
	***P. obliquata* (Blume) Mett.**	1	1	1	1	CO	1	0	0	0	0	0	0	0	0	0	0	0	1	1	2	1
	*P. pectinata* T.Moore	0	0	0	0	NA	NA	0	0	0	0	0	0	0	0	0	0	0	0	0	0	1
	***P. urceolatis* (Hayata) Copeland**	0	1	1	1	NA	0	0	0	0	0	0	0	0	0	0	0	0	0	1	1	0
***Radiogrammitis* Parris**	***R. alepidota* (M.G.Price) Parris**	0	1	1	1	NA	0	0	0	0	0	0	0	0	0	0	0	0	0	1	1	0
	*R. beddomeana* (Alderw.) Parris	0	0	0	0	NA	NA	0	0	0	0	0	0	0	0	0	0	0	0	0	0	1
	***R. ilanensis* T.C.Hsu**	0	0	0	1	NA	0	0	0	0	0	0	0	0	0	0	0	0	0	1	1	0
	*R. jagoriana* (Mett. Ex Kuhn) Parris	0	0	0	0	NA	NA	0	0	0	0	0	0	0	0	0	0	0	0	0	0	1
	***R. moorei* Parris & Ralf Knapp**	0	1	1	1	NA	0	0	0	0	0	0	0	0	0	0	0	0	0	1	1	0
***Radiogrammitis* Parris**	***R. setigera* (Blume) Parris**	1	1	1	1	SY	1	0	0	0	0	0	0	0	0	0	0	0	0	1	1	0
	***R. subnervosa* T.C.HSu**	0	0	0	1	NA	0	0	0	0	0	0	0	0	0	0	0	0	0	1	1	0
	***R. taiwanensis* Parris & Ralf Knapp**	1	1	1	1	MI	0	0	0	0	0	0	0	0	0	0	0	0	0	1	1	0
***Scleroglossum* Alderw.**	***S. pusillum* (Blume) Alderw.**	1	0	0	0	NA	1	0	0	0	0	0	0	0	0	0	0	1	0	0	1	1
	*S. pyxidatun* Alderw.	0	0	0	0	NA	NA	0	0	0	0	0	0	0	0	0	0	0	0	0	0	1
	***S. sulcatum* (Kuhn) Alderw.**	0	1	1	1	MI	1	0	0	0	0	1	1	0	0	0	0	0	1	1	4	1
***Themelium* (T.Moore) Parris**	***T. blechnifrons* (Hayata) Parris**	1	1	1	1	MI	0	0	0	0	0	0	0	0	0	0	0	0	0	1	1	0
	*T. halcoense* (Copel,) Parris	0	0	0	0	NA	NA	0	0	0	0	0	0	0	0	0	0	0	0	0	0	1
	***T. tenuisectum* (Blume) Parris**	1	1	1	1	CO	1	0	0	0	0	0	0	0	0	0	0	0	0	1	1	0
***Tomophyllum* (E.Fournier) Parris**	***T. donianum* (Spreng.) Fraser-Jenk.**	1	1	1	1	MI	0	1	0	0	0	0	0	1	1	1	1	1	0	0	6	1
	*T. repandulum* (Mett.) Parris	0	0	0	0	NA	1	0	0	0	0	0	0	0	0	0	0	0	0	0	0	1
***Xiphopterella* Parris**	***X. devolii* S.J.Moore, Parris, W.L.Chiou**	0	1	1	1	NA	1	0	1	0	1	1	1	0	0	0	0	0	0	1	5	0
	*X. parva* Parris	0	0	0	0	NA	NA	0	0	0	0	0	0	0	0	0	0	0	0	0	0	1
**Species per provinces**		22	31	31	36		18	1	4	2	4	8	9	4	4	2	2	4	10	28		
**Proportion of species diveristy**							46%	3%	11%	5%	11%	22%	24%	11%	11%	5%	5%	11%	27	76%		

Here, we test the hypothesis that conflicting species identity of the Chinese occurrences of *Scleroglossum* reflects the occurrence of two instead of one representative of this genus in mainland China. Both species considered to occur in China, namely *S. pusillum* and *S. sulcatum*, occur throughout tropical Asia, Malay Archipelago, and the Pacific Islands (Parris et al. 2015, Parris 2018). Their reported range includes Thailand (Lindsay et al. 2009) and Vietnam (Parris et al. 2015). To address this question, we studied living materials of this taxon collected from Hainan and Yunnan. We also compared existing herbarium specimens of known occurrences in Guangdong, Guangxi, Hainan, and Yunnan. Several herbarium collections of *Scleroglossum* from Vietnam were also checked carefully. The comparisons were carried out to detect both phenotypic (morphology) and genotypic (DNA sequences) differentiation between the sampling accessions to recover evidence to support or reject the proposal of two species instead of a single species in mainland China.

## Methods

Previous collections of *Scleroglossum* in China were explored by studying digital images available through the online resources of Chinese herbaria [http://www.cvh.ac.cn] and by visiting the herbaria of Kunming Institute of Botany, Chinese Academy of Sciences (KUN), Yunnan University (PYU), and the South China Botanical Garden, Chinese Academy of Sciences (IBSC). All specimens were re-identified using published identification keys (Parris et al. 2015) and comparison with digital images of types available via Global Plants on JSTOR [https://plants.jstor.org]. We also studied published descriptions and images in floristic treatments recording *Scleroglossum* in China (e.g. Ching et al. 1964, Zhang 2000, Knapp 2011, Zhang 2012, Jiang and Liu 2014, Yan and Zhou 2018).

Fieldwork was carried out to obtain new collections from known localities in Hainan (Mt. Yinggeling) and SE Yunnan. Potential new localities in western Yunnan (Tongbiguan) besides previous reported occurrences were explored to assess the real current distribution range in mainland China (Mt. Laojunshan, Yunnan and Mt. Yinggeling, Hainan). Together with samples from different herbaria, these specimens were studied to determine the phenotypic variation and to re-identify the accessions using determination keys (Parris et al. 2015). Morphological observations were carried out using a microscope for some detailed observations.

Genomic DNA was extracted from the newly collected accessions (Hainan and Yunnan) as well as some herbarium specimens including collections from Vietnam, Guangxi, Guangdong and Yunnan using standard DNA extraction protocols (Liu et al. 2007, Liu 2016). Given the available amount of *rbcL* sequences of grammitid ferns in GenBank (www.ncbi.nlm.nih.gov) and the initial analyses of the variation among available sequences of *Scleroglossum*, we restricted our analyses to the *rbcL* component of the CBOL barcode of land plants (CBOL Plant Working Group 2009). The sequences were obtained using primers and protocols used for grammitid ferns in the past (Ranker et al. 2004). All *rbcL* sequences of grammitid ferns available in GenBank till October 2018 were downloaded and integrated in a single alignment using Mesquite 3.04 (Maddison and Maddison 2018). Newly generated sequences were assembled and then incorporated into the global alignment. The alignment included three species of *Scleroglossum* previously studied, namely the GenBank accessions of *S. pusillum* (KM218812, KY712079 from Malay Peninsula and Thailand respectively), *S. sulcatum* (AY460664, AY460665, KY099861 from Pohnpei and Moorea), and *S. wooroonooran* (F.M.Bailey) C. Chr. (KM218809, KM218810 from Australia) plus newly generated sequences of the Hainan Accession (one location, two specimens) and Yunnan Accession (one location, one specimen). Besides, we obtained sequences of two accessions collected in Vietnam that were deposited at KUN. Unfortunately, most of the herbarium specimens were failed in sequence generation. Appendix 1 provides the information on all newly generated accessions including herbarium voucher, area of origin, and GenBank accession number.

The genetic variations among the *rbcL* sequences were compared pairwise among all available and newly generated accessions of *Scleroglossum* (Table 2) by recording sequence similarity in % and number of substitution events. The phylogenetic relationships were reconstructed using maximum likelihood via PhyML 3.0 (Guindon and Gascuel 2003, Guindon et al. 2010) and Bayesian inference via MrBayes 3.2 (Ronquist et al. 2012). The substitution model was determined using jModeltest 2 (Darriba et al. 2012) with the Bayesian Information Criterion (BIC; Schwarz 1978). The number and kind of parameters (mutation type, gamma and invariable sites) were set as the default in the phylogenetic analyses but the parameter values estimated empirically during the phylogenetic analyses (model selected: GTR+I+G). TRACER 1.5 (Rambaut and Drummond 2009) and FIGTREE 1.4.2 (Rambaut 2014) were used to summarize and visualize the results.

**Table 2.** RbcL sequence variation among *Scleroglossum* samples studied. GenBank accession numbers are given for those available in GenBank, whereas specimens accessions are given for newly obtained sequences, namely YunAcc = Yunnan population, HaiAcc = Hainan populations (all sequences obtained for this population were identical), and two Vietnam accessions (VieAcc1 and VieAcc2). Sequence variation are given as pairwise similarity (upper-right corner, in %) and number of substitution events (lower-left corner). Sequence pairs with a similar >99% are marked in Bold.

	YunAcc	HaiAccA&B	KY712079	KM218812	VieAcc1	VietAcc2	AY460664	AY460665	KY099861	KM218809	KM218810
YunAcc	–	98.9	**99.9**	**99.3**	98.5	98.5	98.4	98.5	98.5	98.4	98.5
HaiAccA&B	13	–	99	98.7	98.1	98.1	98.1	98.3	98.3	98.1	98.3
KY712079	1	12	–	**99.4**	98.6	98.7	98.5	98.7	98.7	98.4	98.6
KM218812	9	16	8	–	98.5	98.6	98.6	98.7	98.7	98.1	98.4
VieAcc1	19	24	17	18	–	**99.9**	**99.5**	**99.8**	**99.8**	97.7	97.8
VieAcc2	18	23	16	17	1	–	**99.7**	**99.8**	**99.8**	97.7	98
AY460664	20	23	18	17	6	4	–	**99.8**	**99.8**	97.7	98
AY460665	18	21	16	15	3	2	2	–	**100**	97.8	98.1
KY099861	18	21	16	15	3	2	2	0	–	97.8	98.1
KM218809	20	24	20	24	29	29	29	27	27	–	**99.4**
KM218810	18	21	17	20	27	26	26	24	24	7	–

The biogeography of the Chinese grammitid ferns (Table 1) was assessed based on existing records reported in recent treatments combined with herbaria records. The distribution was then explored using neighbor-joining cluster analyses reconstructed with Jaccard distances. The dataset was also used to assess the conservation status based on the IUCN Red List Categories and Criteria (IUCN 2012). All statistical analyses were carried out using R version 3.5.1 (R Development Core Team 2018). The IUCN assessment was assisted by the ConR package (Dauby et al. 2017).

## Results

### Phenotypic differentiation

**Figure 1.**
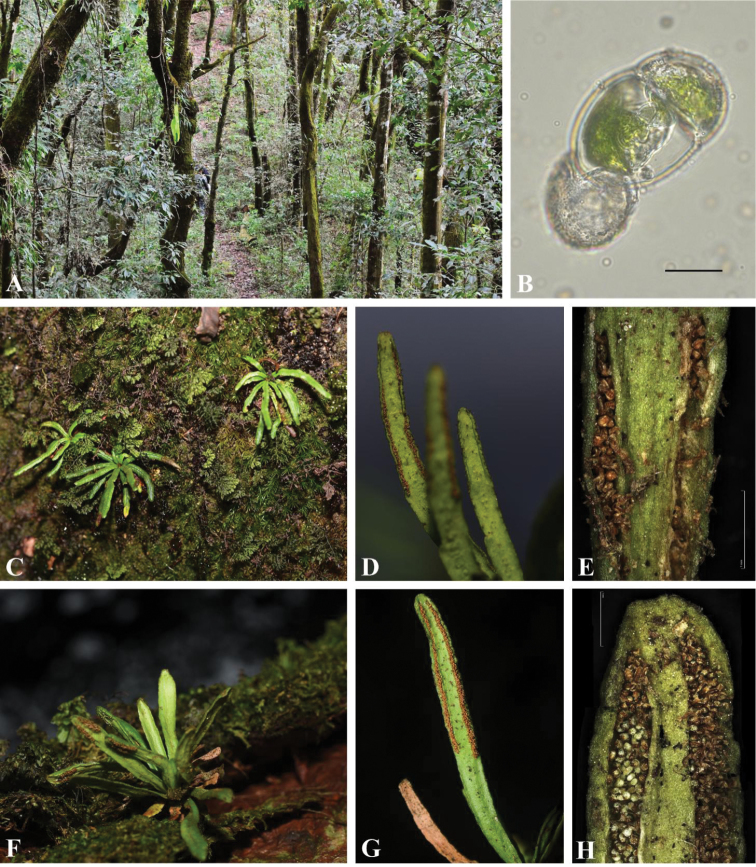
The grammitid genus *Scleroglossum* in China. **A–E***Scleroglossum pusillum* in Yunnan (YunAcc) and **F–H***Scleroglossum sulcatum* in Hainan (HaiAcc). **A** habitat of *S. pusillum* occurrences in Yunnan **B** germinated green spore recovered from opened mature sporangia of *S. pusillum*. The remains of the spore wall are visible at the lower part of the larger of the two cells that both contain mature chloroplasts. The smaller cell is the first daughter cell formed by the first cell division **C** habit of *S. pusillum***D** close up of the dorsal surface of the simple leaves showing the location of the submarginal sori and the occurrences of brown branched hairs of *S. pusillum***E** close up of the sorus structure showing the placement at the submargin of the leaves in *S. pusillum*. **F** habit of *S. sulcatum***G** close up of the dorsal surface of the simple leaves showing the location of the not submarginal sori and the occurrences of brown branched hairs of *S. sulcatum***H** close up of the sorus structure showing the placement of the sori in dorsal grooves and a distinct lamina margin in *S. sulcatum*. Scale bars: 0.02 mm (**B**); 1 mm (**E, H**).

New locality in Yunnan is occurred in Tongbiguan Provincial Nature Reserve (Dehong, 24°19'55.68"N, 97°43'45.51"E, alt., 1443m). The Yunnan accession (Fig. 1A–E) shared with the Hainan accession (Mt. Yinggeling, 19°24'15.9"N, 109°32.07'E, alt., 1749m; Fig. 1F–H) the characters of short, erect, radial rhizome (Fig. 1C, 1F), non-articulated leaves with simple lamina and short petioles, concolorous and glabrous rhizome scales, free veins, absence of hydathodes, branches hairs attached to the lamina (Fig. 1D, 1G), and coenosori arranged in parallel to the margins (Fig. 1E, 1H). However, the Yunnan accession differed from the Hainan accession in the lamina texture and positioning of the coenosori. The Yunnan accession (YunAcc) has a thick lamina margin with the coenosori arranged in submarginal grooves (Fig. 1E), whereas the Hainan accession (HaiAcc) shows a thin lamina margin with the coenosori arranged in grooves on the abaxial surface of the lamina (Fig. 1H). Compared with the established species differentiation in the genus *Scleroglossum*, the Hainan accession should be identified as *S. sulcatum*, whereas the Yunnan accession should be identified as *S. pusillum*. The Yunnan accession differs from *S. pyxidatum* because the lamina was not narrowed below the sori and wider than 2 mm.

### Genotypic differentiation

Pairwise comparisons of the *rbcL* sequences of the seven distinct *Scleroglossum* sequences recovered a sequence variation between 99.7% and 100% (Table 2). The two accessions of *S. wooroonooran* (KM218809, KM218810) showed a similarity of 99.4%, whereas the accessions of *S. pusillum* from Malay Peninsula (KM218812) and Thailand (KM211812) showed a similarity of 99.4%. The accession obtained in Yunnan (YunAcc in Table 2) showed a similarity of 99.9% with the *S. pusillum* from Thailand and a 99.3% similarity with *S. pusillum* of the Malay Peninsula. The Hainan accession (HaiAcc) and Yunnan accession (YunAcc) showed a similarity of 98.9%. Accessions of *S. sulcatum* obtained from Pacific Islands and Vietnam showed a similarity between 100% and 99.5%. The Yunnan accession and Hainan accession showed similarities to accessions of *S. sulcatum* below 99.0% (Table 2).

**Figure 2.**
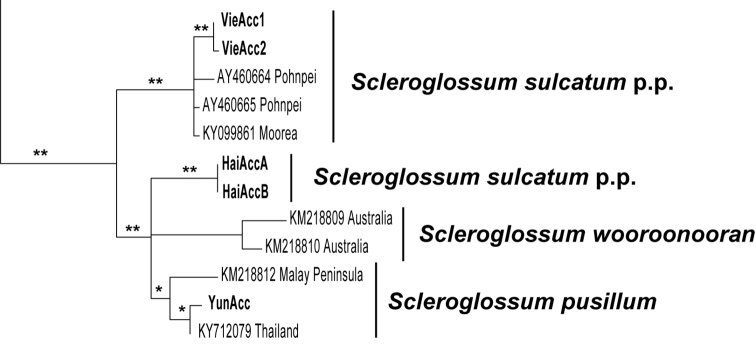
Reconstruction of the phylogenetic relationships of *Scleroglossum* species occurring in China including all accessions available in GenBank (October 2018). Newly generated accessions are printed in bold: HaiAcc = Hainan Accessions, two sequences obtained from individual A and B; YunAcc = Yunnan Accessions; VieAcc1 and VieAcc2 = two accessions obtained from the herbaria collections at KUN representing two independent collections (see Suppl. material 1: Table S1). The areas of origin are given for *Scleroglossum* GenBank accessions. The presented consensus phylogram is based on a Bayesian inference of Phylogeny including 690 accessions represented by *rbcL* sequences. Clades composed by non-*Scleroglossum* accessions were pruned. Two stars mark clades with a posterior confidence of p = 1.00 whereas one star marks clade with a posterior confidence p ≥ 0.95 and < 1.00. p.p. = pro parts to indicate the polyphyly of *S. sulcatum*.

The phylogenetic reconstruction recovered the Yunnan accessions to be nested in clade comprising the two *Scleroglossum pusillum* accessions (Fig. 2), whereas the Hainan accessions formed an independent lineage that was part of the polytomy involving the *S. pusillum* and *S. wooroonooran*. The Vietnam accessions nested together with previously obtained accessions of *S. sulcatum* occurring on the Pacific Islands (Moorea, Pohnpei).

## Discussion

### Species identity

The Hainan and Yunnan accession are distinct in both phenotype and genotype. Thus, two instead of one species of *Scleroglossum* occur in mainland China. Both genotypic and phenotypic evidence support the conclusion that the Yunnan accessions belong to *S. pusillum*. This is consistent with the original report of this taxon in Yunnan (Lu and Zhang 1994). Phylogenetic analyses supported *S. pusillum* occurring in Thailand as the closest relative of the Yunnan occurrences. In contrast, the taxonomic treatment of the Hainan accession is less clear. Phenotypic evidence supports the treatment as *S. sulcatum*, as established previously in the FOC treatment (Moore and Parris 2013). However, the genotypic evidence did not support this conclusion because the Hainan accessions were distinct from all other accessions obtained so far for *Scleroglossum* including not only *S. pusillum* (KM218812; KY712079) and *S. wooroonooran* (KM218809, KM218810), but also *S. sulcatum*. The phylogenetic analyses recovered a clade comprising accessions with the *S. sulcatum* morphology collected in the Pacific Islands of Moorea (KY099861) and Pohnpei (AY40664, AY40665) as well as the two newly obtained accessions from Vietnam. Thus, the Hainan accession may represent a new species which is morphological distinct from the Yunnan accession but not from the *S. sulcatum* accessions from Vietnam. Morphological evidence supports the occurrence of the *S. sulcatum* morphotype also in Guangxi, Guangdong, and Taiwan. However, we currently lack genotypic evidence to assign these specimens to the genotypic differentiated forms of *S. sulcatum*.

### Taxonomic treatment

Species of *Scleroglossum* occurring in mainland China can be identified using the morphological key presented for Vietnam species (Parris et al. 2015). However, we encountered a conflict between the phenotypic and genotypic variation because *S. sulcatum* was recovered as polyphyletic (Fig. 2). This conflict may be explained as a consequence of cryptic speciation (Bickford et al. 2007). Conflicting results between genotype- and phenotype- based species recognition have been reported for several recent diverging fern lineages (Paris et al. 1989) and epiphytic liverworts colonizing similar habitats as *Scleroglossum* (Yu et al. 2013). However, the taxonomic treatment of cryptic or semi-cryptic species is challenging as a consequence of theoretical and practical issues (Jörger and Schrödl 2013). Given the small sample size, we recommend a treatment that is based on the phenotypic variation, meaning *S. sulcatum*, until more evidence will be obtained. This approach avoids unstable taxonomic solutions which are important especially given the lack of evidence concerning the genotype of the type collection of this widespread species. The type was collected on the island of Sri Lanka (lectotype: Thwaites 3807; see Parris et al. 2015) however none of the currently available *rbcL* sequences of this species has been obtained from Sri Lanka. Given the cryptic phenotypic differentiation of the two genotypes, we cannot conclude to which the type may belong. Thus, the introduction of a new name may create more confusion but we must stress that the taxonomic status of the populations currently treated as *S. sulcatum* require further study.

### DNA barcoding

Besides this problem, DNA barcoding using the *rbcL* region appears to be sufficient to resolve the DNA based identification of grammitid ferns in China with all species included having a distinct *rbcL* sequence. However, several species such as the Hainan endemic *Oreogrammitis hainanensis* Parris require to be studied to confirm that this conclusion is true for all grammitid ferns occurring in China. So far, *rbcL* sequences have been obtained for only 45.9% of the Chinese grammitid species. Representatives of the four genera including more than one species in China that were presented with at least two species – namely *Calymmodon* C. Presl, *Micropolypodium* Hayata, *Oreogrammitis* Copel., and *Prosaptia* C. Presl – were distinct from each other in our dataset. Some of the Chinese species were not clearly distinct from closely related non-Chinese species, e.g. *P. contigua* and *P. obliquata*. A further limitation of *rbcL* based barcoding is the problem differentiating some of the proposed generic concepts, such as *Oreogrammitis* Copel., *Radiogrammitis* Parris, and *Themelium* (E. Four.) Parris (see also Sundue et al. 2014). The generic concept of these ferns requires arguably further attention besides the urgent need to focus more on the species taxonomy.

In turn, the application of DNA barcoding recovered a conflict between taxonomic treatments based exclusively on phenotypic evidence with the genotypic evidence. This is consistent with the hypothesis that the frequent employment of these approaches will not only help to resolve conflicting interpretations of phenotypic differentiation (see Yu et al. 2013, Zhou et al. 2016) but also recover more cases of cryptic/ semi-cryptic species differentiation (Pickford et al. 2006). Given the species richness of the fern floras of the southern provinces of China, future studies integrating phenotypic and genotypic evidence may recover more instances of cryptic or semi-cryptic taxa. Some of these taxa may turn out to be the consequence of hybrid-speciation (e.g. Liu et al. 2018) but other processes such as limitations to the accessible morphospace may contribute also to the accumulation of cryptic fern diversity (see Schneider 2016). The genus *Scleroglossum* as well as other grammitid ferns is arguably well suited to explore the role of rapid diversification and ecological conservatism in the decoupling of the accumulation of species diversity and morphological disparity (Schneider 2016). As shown by Liu et al. (2018), the interpretation of DNA barcode based species identification may result in misleading species assessment if the ploidy level has not been assessed. However, relatively little attention has been given to the study of polyploidy in grammitid ferns so far. About 23% of the published 43 chromosome counts of grammitid ferns indicate the occurrence of polyploidy (Schneider unpublished), which is substantially lower than the frequency of polyploidy in *Asplenium* (Schneider et al. 2017). However, no chromosome counts have been published for the genus *Scleroglossum* yet.

The recovered success of DNA based identification of *Scleroglossum* species may enable future studies to report not only the distribution of the sporophytic generation but also the distribution of the gametophytic stage of the life cycle. A recent study provides distinct distribution patterns observed for fern gametophyte and sporophyte generations of the same species on the Pacific island of Moorea (Nitta et al. 2017). This is especially important to studies focusing on grammitid ferns because this fern lineage includes gametophytes reproducing independently of their sporophytes in geographically disjunct populations (Farrar 1967; Dassler and Farrar 2001).

### Biogeography of Chinese grammitid ferns

Given the global distribution of grammitid ferns (see Sundue et al. 2014; Bauret et al. 2017), Chinese grammitid ferns occur mainly in the two larger islands namely Hainan (10 spp., 27% of the Chinese species diversity) and Taiwan (28 spp., 76% the Chinese diversity) and preferably in the southern provinces of Guangdong (8 spp., 22% the Chinese diversity) and Guangxi (9 spp., 24% the Chinese diversity). The remaining provinces with wet subtropical to tropical climate house four (Fujian, Guizhou, Hunan, Yunnan, Zhejiang), two (Jiangxi, Sichuan, Xizang), or one (Anhui) species (Table 1). The low number of grammitid species recorded until now in Yunnan is a bit surprising given the remarkable fern richness of this province especially in the tropical southern parts (Zhou et al. 2016).

**Figure 3.**
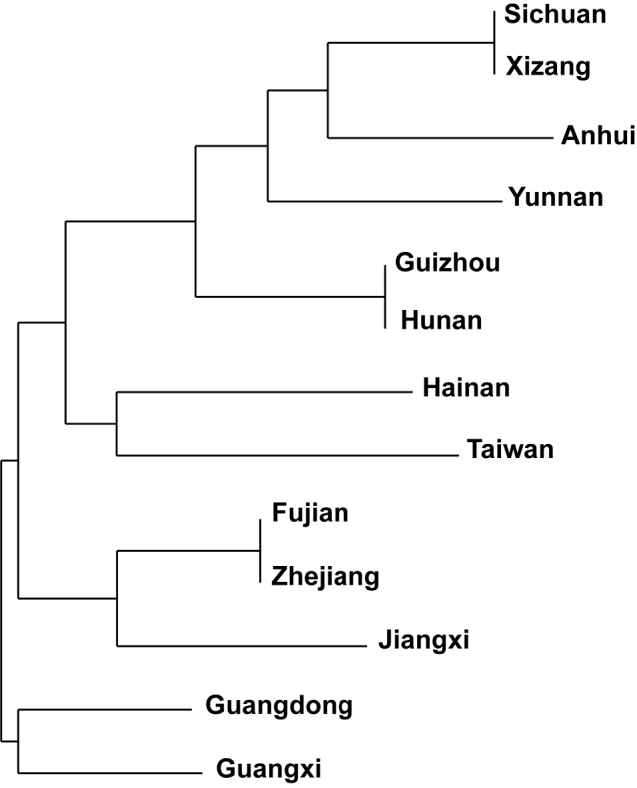
Similarity clustering of the Chinese provinces that are home to at least one grammitid ferns. The clustering analysis was carried out using Jaccard distances and the NJ-tree building algorithm. The underlying scoring (Table 1) was carried out as absence (0) and presence (1).

Analyses focusing on the similarities among the grammitid floras of Chinese provinces recovered similarities (Fig. 3) between the grammitid rich islands of Hainan (10 spp.) and Taiwan (28 spp.) and the two southern provinces Guangdong (8 spp.) and Guangxi (9 spp.). The East China provinces Fujian (4 spp.), Zhejiang (4 spp.), and Jiangxi (2 spp.) formed together a cluster (Fig. 3), whereas Anhui (1 spp.) was nested in cluster including Southwest China provinces, namely Sichuan (2 spp.), Xizang (2 spp.), Yunnan (4 app.), Guizhou (2 spp.), and Hunan (2 spp.). These results are consistent with the prediction that the grammitid diversity is mainly shaped by differences in the climatic conditions but this requires further analyses.

### Conservation of *Scleroglossum* in China

Several species of grammitids including *Scleroglossum sulcatum* were listed in the Red List of Chinese Plants (Qin et al. 2017), but none of the Chinese species have been listed in the list of rare and threatened species of Asia (Ebihara et al. 2012). Furthermore, the more recent list did not mention any species of *Scleroglossum* as rare or threatened. This difference is arguably the consequence of the context differences of a national (Qin et al. 2017) and a global (Ebihara et al. 2012) focus of the assessment. As a consequence of our results, the Red List of Chinese Plants needs some changes. In 2017, *S. sulcatum* was listed as VU D1+2 (Qin et al. 2017), but this assessment is likely based on the assumption of a single species with occurrences in five provinces of China (Yan et al. 2016). Given the recognition of the Yunnan occurrences as *S. pusillum*, the range of *S. sulcatum* has been reduced from five to four provinces of China. However, the current range is sufficient to maintain the status reported (Qin et al. 2017). In contrast, the Chinese populations of *S. pusillum* may be highly vulnerable but further assessments are needed. We recorded this species for the first time to occur in western Yunnan namely Mt. Tongbiguan, Dehong but were unable to confirm the previous records in southeastern Yunnan namely Mt. Laojunshan, Malipo. However, both species are considered as least concern (LC) considering the distribution range comprising most of tropical Asia and some islands in the Pacific and Indian Oceans.

In our study, we are able to confirm the occurrences in both Yunnan and Hainan. As mentioned above we obtained a new record from western Yunnan but failed to reconfirm the occurrence in southeastern Yunnan. During fieldwork we confirmed the occurrence of these ferns in one of the two locations recorded in Hainan namely the Mt. Yinggeling population but did not recollect the Mt. Wuzhishan population. The Guangxi and Guangdong occurrences have been surveyed in recent years (Zhou and Li 1993, Yan et al. 2007, Zhang and Li 2011, Jiang and Liu 2014). Thus, all recorded populations of these ferns in China are arguably stable despite the restriction to isolated localities result in local threats. All known Chinese occurrences are within national parks that will arguably enable effective protection of these populations. However, we still recommend that the number of individuals need to be regularly assessed. At the current stage, the IUCN red list assessment are based on species identification using the phenotypic differentiation only and thus they are arguably misleading for lineages including cryptic- or semi-cryptic species as recovered in *S. sulcatum* (see above). Thus, assessments of the distribution of *S. sulcatum* are arguably inaccurate. Furthermore, very little information exists about the number of individuals per site which limits any assessment of the potential dynamics of population size shrinking or expansion. Finally, the distribution of the two *Scleroglossum* species throughout tropical SE Asia and Pacific Islands may lack the accuracy requested to enable reliable IUCN assessments. Many older collections of these species lack detailed information about the collecting sites and several putative occurrences require to be re-confirmed by recollecting.

## Conclusions

Our study on the grammitid fern *Scleroglossum* in China illustrated the importance of combining field assessments together with traditional morphological (phenotype) and DNA barcoding (genotype) approaches to improve our knowledge of the distribution of plants occurring in remote wet forest habitats of southern China. The assessment and conservation of these local rare species is challenging but the usage of DNA barcoding approaches may enable reliable surveys that do not require the involvement of the few taxonomic experts. In turn, these assessments may also provide crucial information to improve existing taxonomic treatments by providing evidence to test taxonomic concepts that may be challenged by the occurrence of cryptic species.

## Supplementary materials

Supplementary material 1 Table S1 Data type: Collection data https://binary.pensoft.net/file/327874This dataset is made available under the Open Database License (http://opendatacommons.org/licenses/odbl/1.0/). The Open Database License (ODbL) is a license agreement intended to allow users to freely share, modify, and use this Dataset while maintaining this same freedom for others, provided that the original source and author(s) are credited.Hong-Mei Liu, Jian-Yong Shen, Zhen-Long Liang, Feng Peng, Wei-Zhen Wang, Zu-Wei Yang, Shuang Wang, Barbara Parris, Harald Schneider
